# Protegrin-1 Regulates Porcine Granulosa Cell Proliferation *via* the EGFR-ERK1/2/p38 Signaling Pathway *in vitro*

**DOI:** 10.3389/fphys.2021.673777

**Published:** 2021-05-21

**Authors:** Bo Pan, Canying Liu, Xiaoshu Zhan, Julang Li

**Affiliations:** ^1^Department of Animal BioSciences, University of Guelph, Guelph, ON, Canada; ^2^Department of Life Science and Engineering, Foshan University, Foshan, China

**Keywords:** protegrin-1, granulosa cells, ERK1/2 signaling pathway, p38 signaling pathway, EGFR, proliferation

## Abstract

Antimicrobial peptides (AMPs) are traditionally known to be essential components in host defense *via* their broad activities against bacteria, fungi, viruses, and protozoa. Their immunomodulatory properties have also recently received considerable attention in mammalian somatic tissues of various species. However, little is known regarding the role of AMPs in the development and maturation of ovarian follicles. Protegrin-1 (PG-1) is an antimicrobial peptide which is known to have potent antimicrobial activity against both gram positive and negative bacteria. Here we report that the PG-1 is present in the porcine ovarian follicular fluid. Treatment of granulosa cell with PG-1 enhanced granulosa cell proliferation in a dose-dependent manner. This is accompanied by increased expression of cell-cycle progression-related genes such as cyclin D1(*CCND1*), cyclin D2 (*CCND2*), and cyclin B1(*CCNB1*). Additionally, Western blot analysis showed that PG-1 increased phosphorylated epidermal growth factor receptor (EGFR), and the phosphorylated-/total extracellular signal-regulated kinase (ERK)1/2 ratio. Pretreatment with either U0126, a specific ERK1/2 phosphorylation inhibitor, or EGFR kinase inhibitor, AG1478, blocked the PG-1 induced proliferation. Moreover, luciferase reporter assay revealed that ETS domain-containing protein-1 (Elk1) C/EBP homologous protein (CHOP), and the transcription activators downstream of the MAPK pathway, were activated by PG-1. These data collectively suggest that PG-1 may regulate pig granulosa cell proliferation via EGFR-MAPK pathway., Hence, our finding offers insights into the role of antimicrobial peptides on follicular development regulation.

## Introduction

Antral follicle is characterized by a fluid-filled cavity, also termed as antrum adjacent to the oocyte and the surrounding granulosa cells. Ovarian follicle structure changes dramatically, the change in its structure reveals that proliferating granulosa cells support the progression of follicular growth and formation of the antrum. It is believed that follicular fluid components originate from granulosa cells, thecal cells, oocytes, and blood plasma composition transferred through the thecal capillaries (Hirshfield, 1991; Kotsuji and Tominaga, 1994; Maruo et al., 1999; Rodgers et al., 2003; Komatsu and Masubuchi, 2017). The rapid accumulation of FF and granulosa cell divisions lead to the size increase of small antral follicles, which is a necessary process of follicular development (Fortune, 1994). The FF compartment has a remarkable diversity of biomolecules, including steroid hormones, metabolites, polysaccharides, proteins and small peptides, which all play a role in granulosa cell function, oocyte quality, and success of fertilization (Fortune, 1994; Ambekar et al., 2013; Basuino and Silveira, 2016; Kushnir et al., 2016; Shen et al., 2017).

Granulosa cell proliferation is closely related to the cell cycle process which is tightly controlled by cyclin-dependent kinases (Cdks) and their interactions with cyclins and Cdk inhibitors (Golias et al., 2004). These Cdks and Cdk inhibitors also play a role in the regulation of granulosa cell proliferation.

MAPK signaling is one of the signaling pathways that have been identified to regulate granulosa cell functions, and to support the development and maturation of oocytes. The MAPK signaling pathway transfers extracellular stimulus signals to the cytoplasm and nucleus, which activates several transcription factors including Elk1 (ERK pathway), CREB (p38 pathway), c-Jun (JNK pathway), also, alternatively, via secondary signaling, CHOP (p38 pathway), to regulate many cell functions (Wang and Ron, 1996; Vaishnav et al., 2003; Weston and Davis, 2002; Roskoski, 2012). The importance of ERK pathway in granulosa cell function and ovary follicular development has been reported (Ryan et al., 2008). Inhibition of ERK reduced gonadotropin- and IGF-stimulated hormone production by granulosa and theca cells *in vitro*, while *in vivo* inhibition of ERK reduced follicle growth and estradiol production (Ryan et al., 2008). Furthermore, disruption of ERK1/2 in mouse granulosa cells *in vivo* abolishes ovulation, cumulus cell-oocyte complex expansion, oocyte maturation, and luteinization (Fan et al., 2009).

Protegrins are a family of cathelicidins that are specially produced in pigs. Five native protegrin sequences (PG-1 to PG-5) containing 16–18 amino acids, respectively, have been identified. Among these PGs, PG-1 attracted more attention due to its stability *in vivo* (Kokryakov et al., 1993; Choi et al., 2014; Shruti and Rajasekaran, 2019). PGs’ antimicrobial function is well-known (Kokryakov et al., 1993), and we have recently reported its immunomodulatory role during inflammation (Huynh et al., 2018; Penney and Li, 2018). The current study intended to investigate the potential role of PG-1 in the regulation of follicular growth, particularly in granulosa cell function. Additionally, the study aimed to also explore the cellular and molecular mechanisms of the regulatory processes in order to elaborate the physiological role of the AMP in the ovary.

## Materials and Methods

### Granulosa Cell Isolation and Culture

All animal procedures were performed in accordance with the guidelines established by and with the approval of the Animal Care Committee at the University of Guelph. The procedure of ovary collection, granulosa cells isolation and culture were described previously (Sun et al., 2018). Briefly, porcine ovaries were collected from prepubertal gilts at Conestoga Meat Packers Ltd., Canada, and were transported to the university laboratory. The ovaries were rinsed three times with sterile 1 × PBS. Granulosa cells were aspirated from small (1–3 mm in diameter, termed as SGC) and large (4–6 mm in diameter, termed as LGC) follicles by using a 20-mL syringe fitted to an 18-gauge needle. The tubes were positioned still on the lab bench for around 10 min and the upper follicular fluid was discarded. The granulosa cell pellets were mixed and washed with a large volume of DMEM/F12 (Gibco, Carlsbad, CA, United States) supplemented with 1 × antibiotic/antimycotic. Cells were dispersed by pipetting and were washed two additional times to get rid of blood cells. Viable cells, determined by trypan blue exclusion, were seeded at 0.6 × 10^6^/mL in 24-well tissue culture-treated plates in DMEM/F12 with 10% FBS (Gibco) and 1 × antibiotic/antimycotic. Cells were cultured in a humidified atmosphere of 5% CO_2_ at 37.5°C. The medium was removed after 24 h, cells were washed with 1 × PBS, and fresh DMEM/F12 with 10% FBS was added to continue the primary culture.

### Cell Proliferation Assay

To identify the effect of PG-1 on granulosa cell proliferation, the number of living cells of post-treatment samples were determined by TC20^TM^ automated cell counter (BioRad) after trypan blue exclusion. Fifty percent confluent granulosa cells in 24-well plates were stimulated with 0, 1, and 10 μg/ml of PG-1 for 24 h, then gently washed once with 500 μl of 1 × PBS, exposed to 100 μl of Trypsin-EDTA; Sigma-Aldrich, St. Louis, MO, United States) at 37°C for 5 min. Digested cells from plates were scraped, collected and enumerated.

Cell viability was also measured using the Alamar Blue cell Viability reagent (Cat. DAL1025, Thermo Fisher Scientific) according to the manufacturer’s instruction. AlamarBlue Cell Viability Reagent is a resazurin-based solution that functions as a cell health indicator by using the reducing power of living cells to measure viability. Upon entering living cells, resazurin is reduced to resorufin, a compound that is red in color and highly fluorescent. Changes in viability can be easily detected using either an absorbance- or fluorescence-based plate reader. Briefly, around 5 × 10^3^ porcine granulosa cells/well were seeded in 96-well plates with complete culture medium for overnight culture. Then, the culture medium was refreshed without FBS for another 8 h. Furthermore, the cells were treated with synthesized mature PG-1 (Shanghai Top-Peptide Biotechnology Co., Ltd, China, the synthesized amino acid sequence of mPG-1: RGGRLCYCRRRF) for 24 h, 10 μl of Alamar Blue cell Viability reagent was added directly to cells in culture medium. The cells were then mixed thoroughly and a homogenous solution was achieved by lightly tapping the plate several times while avoiding bubbles. The cells were incubated in a cell culture incubator for 0.5–4 h at 37°C until the color turned orange. Then an absorbance wavelength value of 570 nm was detected by the Cytation 5 Cell Imaging Multi-Mode Reader (BioTek). The experiments were performed at least three times.

### RNA Isolation, Reverse Transcription and Real Time Quantitative PCR (RT-qPCR)

Total RNA was isolated from fresh GCs or cultured GCs sample by using the Total RNA Purification Kit in accordance with the manufacturer’s instruction (Norgen Biotek, Thorold, Ontario, Canada). The RNaseFree DNase I Kit (Norgen Biotek) was applied to minimize amounts of genomic DNA contamination. RNA quality was measured by using a NanoDrop-8000 (Thermo Fisher Scientific, Waltham, MA) with the 260/280 values are commonly 2. In addition, RNA integrity was confirmed by gel electrophoresis devoid of the genomic DNA contamination. For each sample, 500 ng of total RNA was used, and first-strand cDNA synthesis was carried out using the SuperScript II System according to the manufacturer’s instruction (Life Technologies, Inc.) and conducted in T100TM Thermal Cycler (Bio-Rad). The cDNA was diluted 1:20 for use in real-time PCR. Real-time PCR analysis was performed using the primers shown in Table 1. Real-time RT-PCR was performed on the Bio-Rad CFX ConnectTM Real-Time System (Bio-Rad) using the Sso-AdvancedTM Universal SYBR Green Supermix (Bio-Rad). The PCR reaction consisted of 10 μl of SYBR Green PCR Master Mix, 100 nm of forward and reverse primers, and 2.0 μl of 1:20-diluted template cDNA in a total volume of 20 μl. Samples were mixed thoroughly and quickly centrifuged in the plate. Samples were amplified via q-PCR beginning with 95°C for 2 min, followed by 40 amplification cycles at 95°C for 5 s, 60°C for 20 s, 65°C for 5 s. Melting curve analyses were performed following the RT-qPCR, and the presence of a single peak confirmed the specificity of the PCR amplification products and the PCR product size was confirmed by gel electrophoresis with no visible primer-dimer products. All the primers were designed either to span an intron or to target exon-exon junctions to guarantee our cDNA template was free from contaminating gDNA. Primer efficiencies and PCR amplification efficiencies assay were generated using a serial dilution of cDNA templates with a range from 95 to 105%. Glyceraldehyde 3-phosphate dehydrogenase (GAPDH) and tyrosine 3-monooxygenase/tryptophan 5-monooxygenase activation protein zeta polypeptide (YWHAZ) were used as reference genes, and a geometric mean of their *Ct*-value was used as an internal control to calculate the relative expression level of target gene using the ΔΔCt method (Livak and Schmittgen, 2001; Vandesompele et al., 2002). All real-time PCRs were performed at least three times, and the changes in gene expression were reported as fold increases relative to untreated controls.

**TABLE 1:** List of primers used for quantitative RT-PCR.

mRNA	Primer sequence (5′–3′)	Product size (bp)	GeneBank accession number
*GAPDH*	F: GTTCCAGTATGATTCCACCCACGGCA R: TGCCAGCCCCAGCATCAAAGGTAGAA	147	NM_001206359.1
*YWHAZ*	F: TGATGATAAGAAAGGGATTGTGG R: GTTCAGCAATGGCTTCATC	203	JN007378.1
*CCND1*	F: CACGACTTCATCGAGCACTT R: GTTTGCGGATGATCTGTTTG	71	AK234224.1
*CCND2*	F: CCAACTGGTTGGTGTCACTG R: GCTCTCCGAAGAAAATGCAG	195	NM_001170768.1
*CDK1*	F: GGGTCAGCTCGCTACTCAAC R: AAGTTTTTGACGTGGGATGC	239	NM_001159304.2
*CDK2*	F: GCATCCCAATGTTGTCCG R: GGGGTGCCTTGTCCAGATA	210	NM_001123097
*CCNB1*	F: CCAACTGGTTGGTGTCACTG R: GCTCTCCGAAGAAAATGCAG	148	NM_001170768
*PCNA*	F: TACGCTAAGGGCAGAAGATAATG R: CTGAGATCTCGGCATATACGTG	191	NM_001291925.1
*p27^*Kip1*^*	F: TGCCTTTAATTGGGTCTC R: GTTGGCTCTTTTGTTTTG	158	NM_214316.1
*p21^*cip1*^*	F: CGTCTCAGGAGGACCATGTG R: TGGTAGAAATCTGTCATGCTGGT	136	XM_013977858.2

### Western Blotting Analysis and Antibodies

Total proteins were isolated from GCs, and the lysates in GCs were obtained by lysing cells in Radioimmunoprecipitation assay buffer containing 1 mM of phenylmethylsulfonyl fluoride (Tribioscience Inc., Palo Alto, CA, United States) and 1 × protease inhibitor cocktail (Cedarlane Laboratories Limited, Hornby, Ontario, Canada). Lysed samples, 20 μL of 4 times diluted FF or 5/15/45 ng of PG-1 standard synthesized from Genscript (the synthesized amino acid sequence of PG-1 standard: RGGRLCYCRRRF) were mixed with 4 × laemmli sample buffer (Catalog # 161-0747, BioRad), then boiled for 10 min, and centrifuged for 3 min at 12,000 × g. The equal amounts of supernatant containing the soluble protein were subjected to 10% SDS-PAGE gel. The proteins were then transferred to polyvinylidene difluoride membranes (Millipore, Billerica, MA< United States) by Trans-BlotR Turbo^TM^ transfer system (Cat. 1704150, BioRad). Membranes were blocked in PBS with 5% skim milk powder at 4°C overnights, and then incubated with the primary antibodies. The primary antibodies including rabbit polyclonal ERK2 (K-23) antibodies (Catalog # SC153; Santa Cruz Biotechnology, Santa Cruz, CA, United States), anti-phosphoERK (Thr202/Tyr204) antibody (Catalog #9101; Cell Signaling Technology, Danvers, MA, United States), EGFR antibody (Catalog # SC-373746, Santa Cruz Biotechnology, Santa Cruz, CA, United States), Phospho-EGFR (Tyr1068) Polyclonal Antibody (Catalog # 44-788G, Thermo Fisher Scientific, United States), The anti-PG-1 antibody was produced by Genscript (Catalog #: A418070254, Genscript, New Jersey, United States). The amino acid sequence to customize the anti-PG-1 body was RGGRLCYCRRRFCVCVGR. The secondary antibody that was used in this study was the anti-rabbit IgG horseradish peroxidase (Catalog # 7074; 1:2000; Cell Signaling Technology). For detection of glyceraldehyde-3-phosphate dehydrogenase (GAPDH, used as a protein loading control), the primary antibody against GAPDH (1:10,000; Abcam, Cambridge, MA, United States), followed by incubation with anti-mouse IgG HRP (1:10,000). Proteins were detected by the Clarity Western ECL substrate and imaged with the ChemiDoc XRS+ System (Bio-Rad, Hercules, CA, United States).

### Transient Transfection and Luciferase Assay

Fresh GCs were seeded and cultured in 24-well plates (Eppendorf, 0030722116) and the cell confluence usually reached 50–70% after 24 h of culture. Transfection was performed with Lipofectamine 3000 (Invitrogen, L3000015) according to the manufacturer’s instruction (1:3 ratio of DNA/Lipofectamine). The Stratagene PathDetect Elk1/C-Jun/CHOP/CREB trans-reporting system (Agilent Technologies, Santa Clara, CA, United States) was used to determine the treatment indirectly/directly stimulates Elk1/C-Jun/CHOP/CREB through phosphorylation of ERK1/2/JNK/p38. The Elk1 trans-reporting system package includes pFA2-Elk1/C-Jun/CHOP/CREB (activator plasmid), pFC2-dbd plasmid (negative control plasmid), and pFC-MEKK plasmid (positive control plasmid). Approximately 425 ng of GAL4-luciferase (luciferase reporter plasmid) and 25 ng pRL-TK (control reporter plasmid expressing Renilla luciferase) were co-transfected into the cultured GCs according to kit recommendations. Cells were cultured and grown in Dulbecco modified Eagle medium (DMEM, Invitrogen) supplemented with 10% fetal bovine serum (FBS) in the first 24 h and in medium containing 0.5% FBS for the final 24 h. Then, the DNA complexes were removed by refreshing with the new medium. The cells were starved in DMEM/FBS-free for 20 h before used for PG-1 or vehicle treatment experiments. After 6-h incubations, cells were washed with ice-cold PBS and lysed with 100 μl Passive Lysis Buffer collected and luciferase assays were performed by following the Dual-Luciferase Reporter Assay System instruction (Promega, E1960). Reporter activity was calculated as relative luciferase activity (firefly luciferase/Renilla luciferase) to correct for transfection efficiency and each experiment was performed at least three times.

### Statistical Analyses

Data represent the mean ± SEM of at least three independent experiments. Statistical differences between all groups were determined using a one-way analysis of variance (ANOVA) followed by Tukey’s test for multiple comparisons. Differences between two groups were determined using Student’s *t*-test. Results were considered significant at *P* < 0.05.

## Results

### PG-1 Is Present in Ovarian Follicular Fluid

**FIGURE 1:**
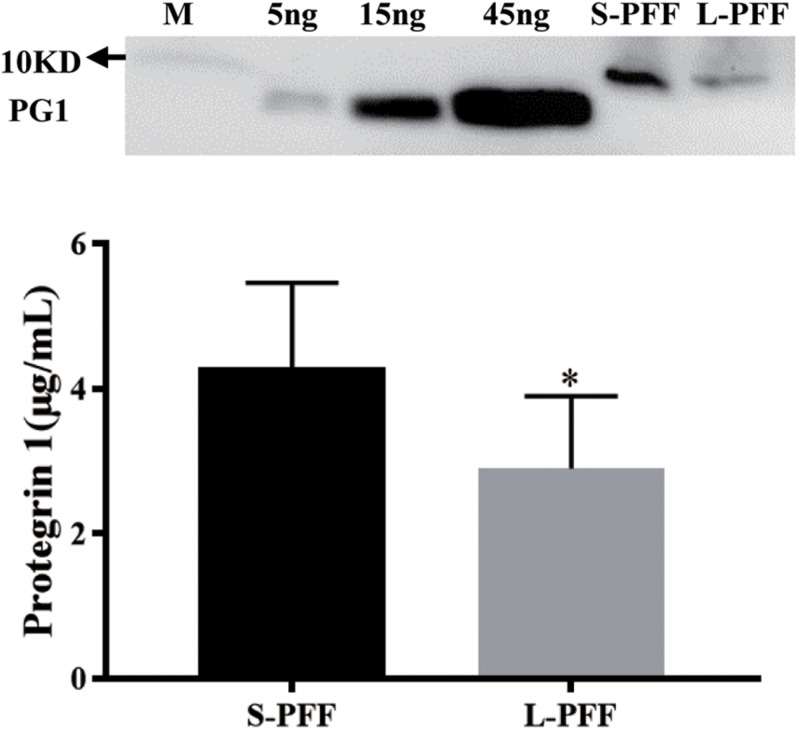
Protegrin 1 is present in follicular fluid. Porcine follicular fluid was isolated from small follicles (S-PFF) and large follicles (L-PFF). Western blot was performed, Data represent the mean ± SEM of three independent experiments *P < 0.05 compared to control.

Porcine follicular fluid from small follicle (1 to 3 mm, S-PFF) and large follicle (4 to 6 mm, L-PFF) were collected and analyzed via Western blots. Using the synthesized PG-1 as a standard, it was estimated that the average concentration of PG1 in S-PFF and L-PFF was (4.29 ± 1.18 μg/ml) and (2.9 ± 1.0 μg/ml), respectively (Figure 1). The length of synthesized PG-1 amino acid sequence (lanes 2–5) used to customize the anti-PG1 antibody is shorter than that of PG-1, thus they migrated faster on the gel than samples from PFF (Figure 1).

### PG-1 Promoted Granulosa Cell Proliferation and Increased Proliferation Related Gene Expression

**FIGURE 2:**
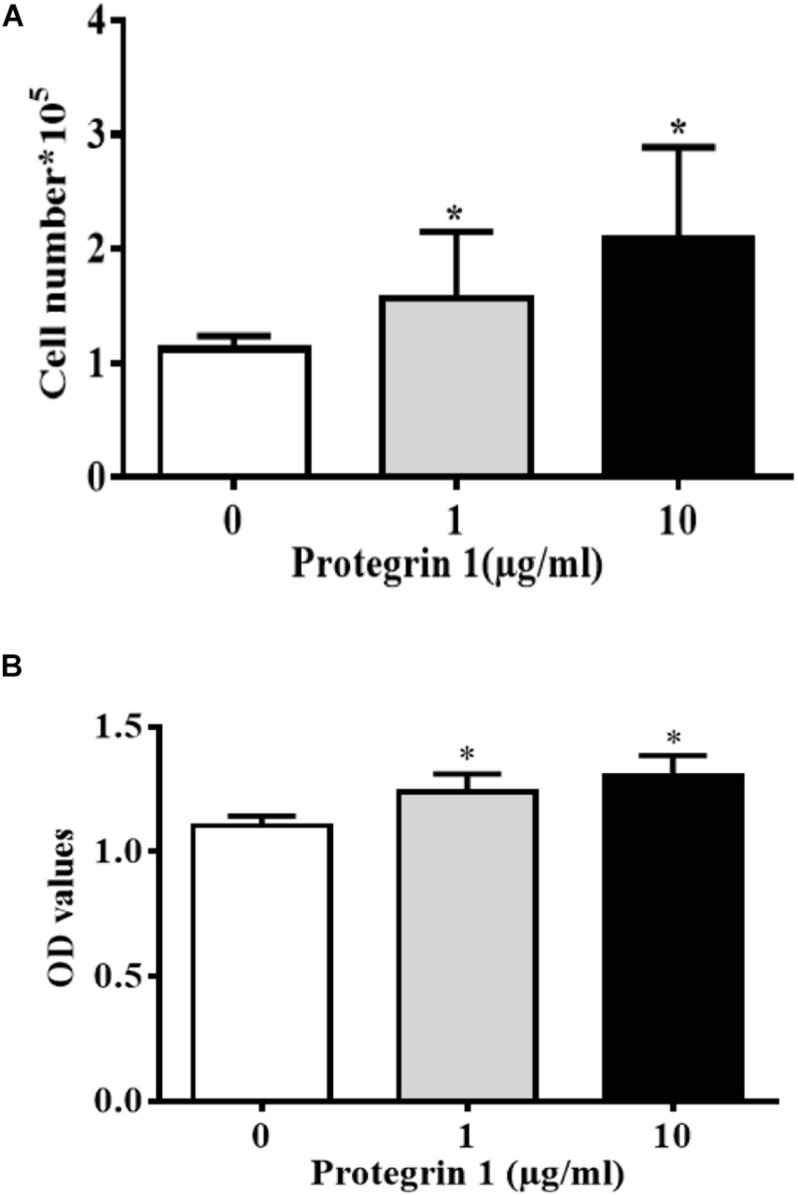
Protegrin-1 promotes granulosa cell proliferation. **(A)** Protegrin-1 increased granulosa cell numbers in a concentration-dependent manner. **(B)** Alamar blue assay results show the proliferation of GCs after treatment of Protegrin-1. Data represent the mean ± SEM of three independent experiments. *P < 0.05 compared to control.

**FIGURE 3:**
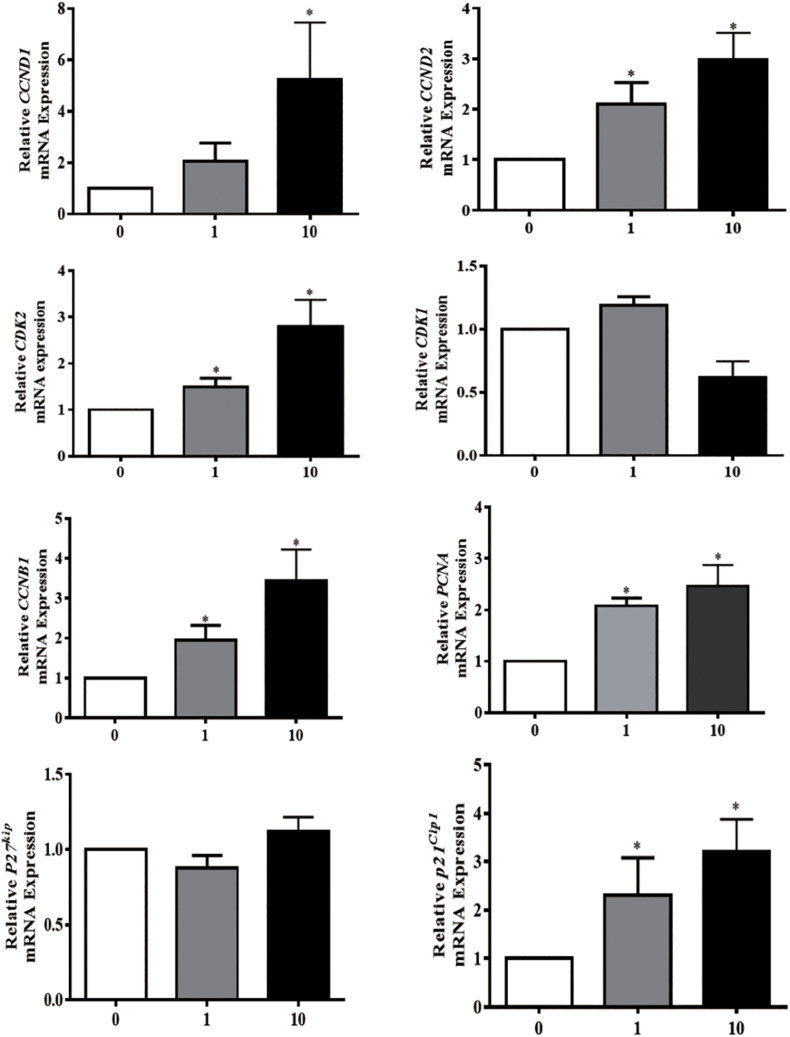
Protegrin-1 increased cell proliferation marker gene expression. Granulosa cells were isolated from small follicles and treated with different concentration of Protegrin-1 for 24 h (0, 1, and 10 μg/ml). Total RNA was isolated, and expression of cell proliferation markers was assessed by RT-qPCR, using both GADPH and YWHAZ as house keeping gene controls. Data represent the mean ± SEM of three independent experiments *P < 0.05 compared to control.

Proliferating granulosa cells support the progression of follicular growth and maturation, We next asked if and how PG-1 influence granulosa cell division. Briefly, SGCs were cultured either in the absence or presence of a various concentrations of PG-1 for 24 h. Cell proliferation was evaluated *via* both cell count analysis using automated cell counter and AlamarBlue assay. At 1 and 10 μg/mL, PG-1 significantly increased granulosa cell numbers (Figure 2A). This concentration-dependent cell proliferation enhancement effect of PG-1 was also confirmed using AlamarBlue assay (Figure 2B). We further examined whether PG-1 regulates the expression of genes associated with granulosa cell proliferation. Consistent with the result on cell proliferation, quantitative RT-PCR analysis revealed that in response to 1 and 10 μg/mL of PG-1, the expression of cyclin D2 (*CCND2*), *CDK2*, cyclin B1 (*CCNB1*), proliferating cell nuclear antigen (*PCNA*), and *P21^*cip1*^* genes that are associated with cell proliferation, were significantly upregulated in cultured SGCs (Figure 3). Meanwhile, the mRNA expression level of cyclin D1 (*CCND1*) is only significantly upregulated at the concentration of 10μg/mL, although PG-1 has no effect on *CDK1* and *P27^*kip*^* mRNA level. The results showed that PG-1 promoted granulosa cell growth and is accompanied by an increase in mRNA levels of proliferation related genes.

### PG-1 May Function via the MAPK/ERK1/2 Pathway in Granulosa Cells

**FIGURE 4:**
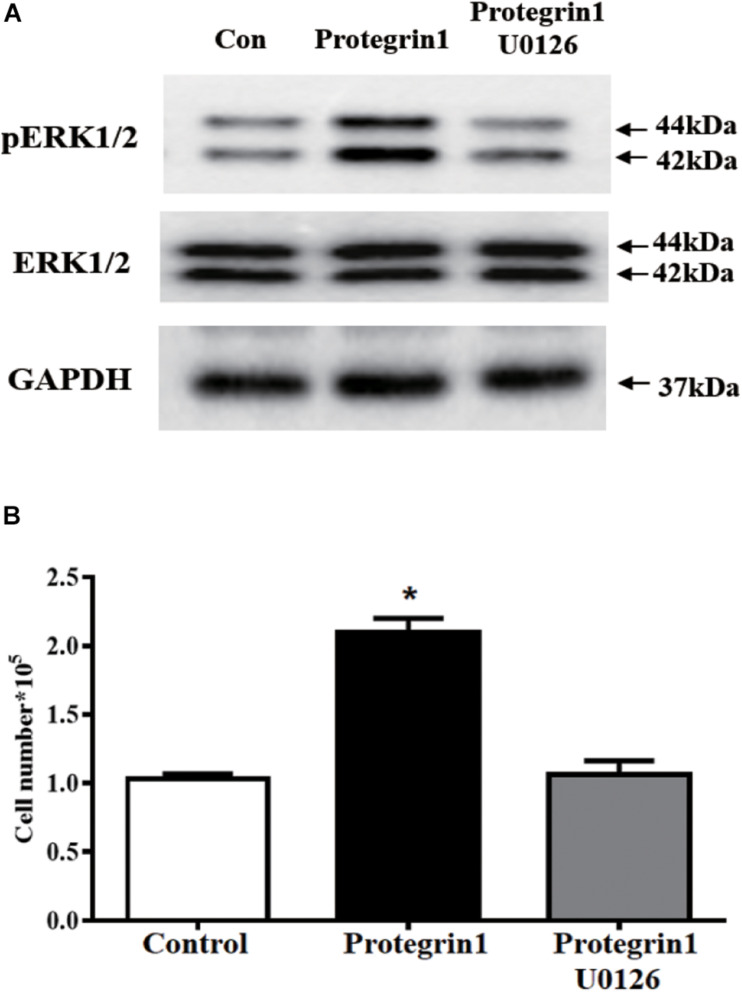
Protegrin-1 increases cell proliferation might via ERK1/2 pathway in cultured granulosa cells. **(A)** Western Blot analysis for relative expression of PERK1/2, ERK1/2, and GAPDH protein. **(B)** pERK1/2 inhibitor U0126 block the Protegrin-1-induced granulosa cell proliferation. Granulosa cells were pretreated with 20 μM U0126 for 30 min, then incubated with 1μg/ml Protegrin-1 for 24 h. Cell proliferation was evaluated by cell counting. Data represent the mean ± SEM of at least three independent experiments *P < 0.05 compared to control.

Mitogen-activated protein kinase (MAPK)/extracellular-signal-regulated kinase (ERK)1/2 pathway was previously reported to be involved in regulating granulosa cell proliferation in other cell type and species (Makarevich et al., 2000; Taniguchi et al., 2004). To test if PG-1 regulates granulosa function through the MAPK/ERK1/2 pathway, western blotting analysis was performed using antibodies against the phosphorylated and unphosphorylated ERK1/2. As shown in Figure 4A, PG-1 increased phosphorylated ERK1/2 but not the total ERK1/2. If PG-1 does induce granulosa cell proliferation via ERK1/2 pathway, one would expect that inhibition of ERK1/2 phosphorylation would reverse its effect. To test this possibility, SGCs were pretreated with U0126 (20 μM), and as expected, PG-1-induced SGCs proliferation was significantly decreased by the ERK1/2 inhibitor (Figure 4B), suggesting that PG-1 induce SGCs proliferation via the ERK1/2 pathway.

**FIGURE 5:**
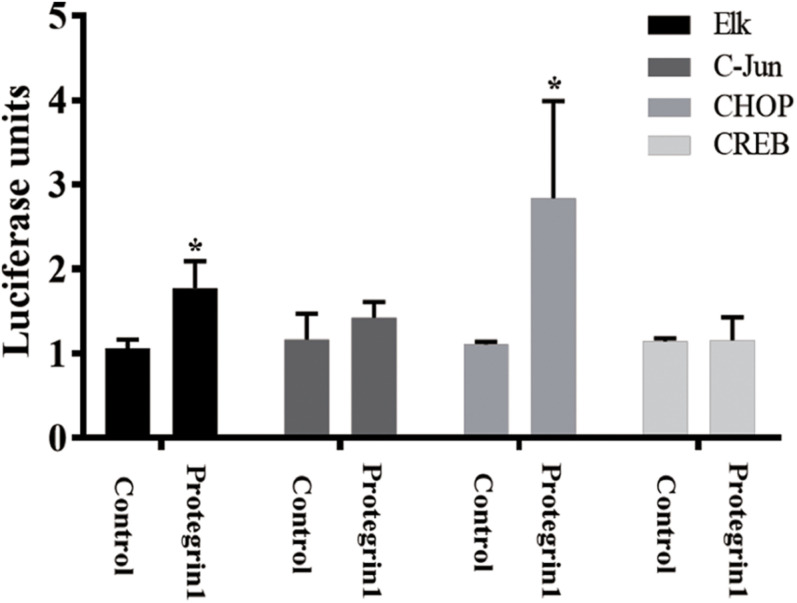
Protegrin-1 activates MAPK signaling pathway. Cultured SGCs were co-transfected with a plasmid, pFR-Luc- a luciferase reporter plasmid, containing a synthetic promoter with five tandem repeats of the GAL4 binding site, and pFA2-CREB, pFA2-Elk1, pFA2-C-Jun, or pFA2-CHOP that expresses the activation domain of each transcription factor fused to the GAL4 DNA binding domain. Eighteen hours after transfection the cells were serum starved and treated with or without Protegrin-1 for 16 h. Luciferase activity, which is indicative of transcription factor dependent activation, is expressed as relative light units compared to control. Data represent the mean ± SEM of three independent experiments *P < 0.05 compared to control.

To verify PG-1 activation of MAPK pathway in granulosa cells, we employed a well-established luciferase-based path-detect system (Agilent Technologies) to monitor activation of transcription factor such as c-Jun, Elk1, cAMP-response element-binding protein (CREB) and CHOP that are downstream components of the MAPK (Wang and Ron, 1996; Weston and Davis, 2002; Vaishnav et al., 2003; Roskoski, 2012). This detection system is an intricate reporter assay that allows for quantifying the activity of the specific transcription factor utilizing the Gal4 DNA binding domain. When the specific pathway is activated, it will result in the activation of the corresponding transcription factor, which in turn stimulates reporter (luciferase) activity. The transcription factor-dependent activation is thus reflected by luciferase activity. As shown in Figure 5, no effect was observed for the activities of c-Jun and CREB, yet PG-1 treatment resulted in an increase of Elk1 and CHOP activities. This data further demonstrated that PG-1 functionally activate the MAPK pathway.

### PG-1 May Function via Epidermal Growth Factor Receptor (EGFR) in Granulosa Cell

**FIGURE 6:**
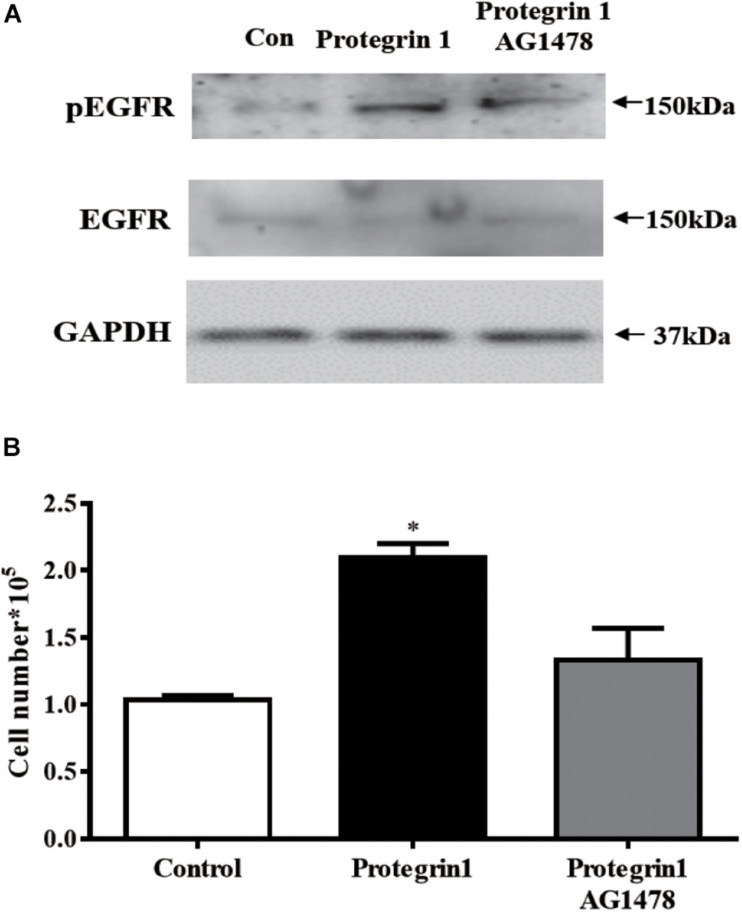
Protegrin-1 increases cell proliferation might via upregulating phosphorylation of EGFR (pEGFR) in cultured granulosa cells. **(A)** Western Blot analysis for relative expression of pEGFR, EGFR and GAPDH protein. **(B)** pEGFR inhibitor AG1478 on Protegrin-1-induced granulosa cell proliferation. SGCs were pretreat with 20 μM AG1478 for 30 min or unpretreat, then incubated with 1 μg/ml Protegrin-1 for 24 h and cell proliferation was evaluated by cell counting. The data shown are representative of three independent experiments. Data represent the mean ± SEM of three independent experiments *P < 0.05 compared to control.

Within MAPK pathway, receptor tyrosine kinase, such as EGFR kinase, acts as an upstream regulator of RAS/RAF/MEK/ERK (Ge et al., 2013). To further investigate if EGFR may be activated by PG-1, we assessed the level of EGFR phosphorylated (pEGFR)–and unphosphorylated-EGFR. Western blotting demonstrated that pEGFR was notably enhanced by PG-1 (Figure 6A), and the enhancement was inhibited by EGFR kinase-specific inhibitor AG1478 (Zhu et al., 2001; Figure 6A), suggesting that PG-1 activated ERK1/2 and p38 pathway via its EGFR phosphorylation. Furthermore, pretreatment of EGFR kinase-specific inhibitor AG1478 also blocked PG-1-induced SGCs proliferation (Figure 6B). Our results support the notion that PG-1 stimulate cell proliferation via the EGFR-ERK1/2/p38 pathway in granulosa cells.

## Discussion

The role and mechanism of PG-1 in pathogen killing and immunomodulation has been well elaborated in previous studies (Knyght et al., 2016; Penney and Li, 2018). However, it was unknown if PG-1 played any role in regulating ovarian function. The current study aimed to test the effects of PG-1 on granulosa cell activity and to identify the potential pathway that is involved.

To our knowledge, this is the first study to report on the presence of PG-1 in the follicular fluid. In our study, the concentration of PG-1 in follicular fluid ranges from 2 to 4 μg/mL. This seems to be in line with the reported physiological concentration of LL-37 (0.5–3 μg/mL in the plasma), a human AMP in the cathelicidin family (Sorensen et al., 1997). It is possible that PG-1 is secreted from the resident neutrophils of the ovary as well as the neutrophils in the blood stream diffuses into the follicular fluid as what has been reported of other blood molecules (Siddiqui et al., 2009). Additionally, neutrophils from the blood are known to be recruited to the ovary to produce bioactive factors there locally, to involve in extracellular metric degradation, follicle maturation, ovulation, luteal formation (Oakley et al., 2010, 2011). Thus, it is also possible that PG-1 is produced locally by these infiltrated leukocytes in the ovary. For example, a previous report suggested that the origin of another AMP human α-defensin in FF is from the resident neutrophils of the ovary (Das et al., 2008).

Granulosa cell proliferation takes part in folliculogenesis and oocyte growth. Our finding on the mitogenic effect of PG-1 suggesting a role of the antimicrobial peptide in this process. Our *in vitro* studies showed that PG-1 stimulated GC proliferation in a dose-dependent manner. Interestingly, PG-1 was also shown to significantly increase intestinal porcine enterocytes migration (Penney and Li, 2018), although PG-1 does not affect the proliferation of intestinal cells (Huynh et al., 2018; Penney and Li, 2018), these findings suggest that the mitogen role of PG-1 maybe cell type specific. Granulosa cell proliferation process is accomplished by the regulated expression of both cyclins and cyclin-dependent kinases (CDKs). Our results showed that when treated with PG-1, the expression of positive regulators of CDK activity and cell cycle progression, *CCND1*, *CCND2*, *CDK2*, *CCNB1*, *PCNA*, and *P21^*cip1*^*, were all enhanced. The up-regulation of *CCND1* mRNA expression is also involved in granulosa cell proliferation induced by the brain-derived neurotrophic factor, β-defensin 3 and fibroblast growth factor 9 (Totty et al., 2017; Chen et al., 2019; Liu et al., 2019). In the ovary, CCND2 is mainly expressed in granulosa cells and is required for granulosa cell proliferation during follicular development (Schwartz and Shah, 2005; Stanley et al., 2011). In the *CCND2*^–/^*^–^* mice, follicles have a reduced number of granulosa cells, remain small and fail to ovulate (Sicinski et al., 1996; Robker and Richards, 1998). Reduction of forskolin-stimulated *CCND2* mRNA expression by 5α-dihydrotestosterone leads to cell cycle arrest resulting in reduced granulosa cell proliferation (Kayampilly and Menon, 2004). CDK2 is dispensable for the mitotic cell cycle and crucial for the first meiotic division of male and female germ cells (Malumbres and Barbacid, 2005). Up-regulation of *CDK2* gene expression was associated with the induced effect on tendon cell proliferation exerted by platelet-rich plasma (Yu et al., 2015). *CCNB1* expression was also increased in the porcine granulosa cell by copper or iron sulfate, which induced cell proliferation and suppressed apoptosis (Kolesarova et al., 2011; Roychoudhury et al., 2014). PCNA is a marker of cell proliferation as it plays an important role in various cellular processes such as DNA replication, chromatin remodeling, chromosome segregation, and cell cycle progression (Mathews et al., 1984; Mailand et al., 2013; Lv et al., 2016). Although p21^cip1^ was previously described as a negative regulator of cell growth by inhibiting CDKs (Yang et al., 2004; Su et al., 2017), there is also evidence that p21^cip1^ has a positive function in the cell cycle progression. For example, the induction of p21^cip1^ gene expression by IGF-1 accompanied with an increase of proliferation in the differentiating chondrogenic cell line MCT and primary mouse chondrocytes (Dupont et al., 2003). A modest increase of p21^cip1^ expression in cancer cells can lead to increase of proliferation at non-lethal doses of chemotherapy (Hsu et al., 2019). Here, we demonstrated for the first time that PG-1 upregulated *p21^*cip1*^* mRNA level and promoted granulosa cell proliferation.

Follicular development and maturation involves complex and precise regulatory mechanisms including both extrinsic and intrinsic signaling pathways. In an attempt to identify the potential pathway(s) involved in the effect of PG-1 on granulosa cells, we investigated the activity of the integral transcription factors using a transcription factor activity reporter system of different MAPK sub-pathways. It was found that the antimicrobial peptide only enhanced the activity of Elk and CHOP. Phosphorylation of nuclear transcription factor Elk is catalyzed by ERK1/2, a protein serine/threonine kinase that participates in the Ras-Raf-MEK-ERK signal transduction cascade (Roskoski, 2012). In porcine granulosa cells, the ERK pathway was activated by β-defensin 3 and vaspin to stimulate proliferation (Kurowska et al., 2019; Liu et al., 2019). CHOP, a member of the C/EBP family of transcription factors, is phosphorylated by p38 MAP kinase to mediate effects of cellular stress on growth and differentiation (Wang and Ron, 1996). Previous literature reports that *in vitro* study using porcine trophectoderm cells showed a promoted effect of insulin-like growth factor I on cell proliferation and migration via p38 signaling pathway (Jeong et al., 2014). Our finding that PG-1 induced phosphorylation of the ERK1/2 and p38 pathway, and regulated granulosa cell proliferation was consistent with this notion. MAPK-ERK1/2 pathway regulates *p21^*cip1*^* gene transcription in the differentiating chondrogenic cell line MCT and primary mouse chondrocytes (Beier et al., 1999). The upregulation of *p21^*cip1*^* mRNA level by IGF-1 was also associated with the MAPK-ERK1/2 pathway in MCF-7 human breast cancer cells (Dupont et al., 2003). These studies, combined with our results, suggest that the MAPK-ERK1/2 pathway was involved in the upregulation of *p21^*cip1*^* mRNA by PG-1 stimulation in SGCs. EGFR signaling is one of the significant receptors that is known to be involved in cell activities. In this study, activation of EGFR was observed during the PG-1 stimulation of granulosa cells and the EGFR tyrosine kinase inhibitor AG-1478 partially blocked PG-1-induced proliferation. These findingssuggest that EGFR may be one of the receptors responsible for the effects of PG-1 on granulosa cell activities. PG-1 was previously reported to interact with insulin-like growth factor receptor to stimulate intestinal cell migration (Penney and Li, 2018). IGF1R is expressed on porcine granulosa cell (Han et al., 2019). Whether IGF1R also participate in the promoted effect of PG-1 on granulosa cell proliferation needs further investigation. Activation of EGFR leads to autophosphorylation of receptor tyrosine kinase that initiates a cascade of downstream signaling pathways including the MAPK pathway (Zandi et al., 2007). The EGFR-ERK pathway has been demonstrated to be involved in porcine cumulus cell expansion (Kim et al., 2020), and also associated with mediating the action of heparin-binding EGF-like growth factor, one of the EGFR ligands, on granulosa cell proliferation (Wang et al., 2007). It was reported that EGF-induced ERK1/2 activation occurs mainly through EGFR activation in porcine renal cell (Dong et al., 2004). It may be noted that, our previous study identified that the ERK1/2 pathway was activated by PG-1 through EGFR to regulate porcine intestinal enterocytes migration (Penney and Li, 2018). These reports, together with our findings suggested that PG-1 regulated granulosa cell proliferation through the EGFR-ERK1/2/p38 pathway.

We previously reported that β-defensin 3, an AMP of the defensin family, plays a role in granulosa cell proliferation and migration (Liu et al., 2019). In addition, β-defensin 1 was reported to possible influence on oocyte quality (Zupin et al., 2019). Our current findings on the role of PG-1 in regulating granulosa function further confirm the additional physiological role of antimicrobial peptides in the ovary beyond the well-known activities in killing bacteria and virus. Our data on the potential pathway PG-1 involved in regulating granulosa proliferation offers insights into understanding their mechanism of actions and understanding their action mechanism in the porcine ovary.

## Data Availability Statement

The original contributions presented in the study are included in the article/supplementary material, further inquiries can be directed to the corresponding author/s.

## Author Contributions

All authors listed have made a substantial, direct and intellectual contribution to the work, and approved it for publication.

## Conflict of Interest

The authors declare that the research was conducted in the absence of any commercial or financial relationships that could be construed as a potential conflict of interest.
